# The Battle between Rotavirus and Its Host for Control of the Interferon Signaling Pathway

**DOI:** 10.1371/journal.ppat.1003064

**Published:** 2013-01-24

**Authors:** Michelle M. Arnold, Adrish Sen, Harry B. Greenberg, John T. Patton

**Affiliations:** 1 Laboratory of Infectious Diseases, National Institute of Allergy and Infectious Diseases, National Institutes of Health, Bethesda, Maryland, United States of America; 2 Department of Medicine and Microbiology and Immunology, Stanford University, Stanford, California, and VA Palo Alto Health Care System, Palo Alto, California, United States of America; University of Alberta, Canada

## Abstract

Viral pathogens must overcome innate antiviral responses to replicate successfully in the host organism. Some of the mechanisms viruses use to interfere with antiviral responses in the infected cell include preventing detection of viral components, perturbing the function of transcription factors that initiate antiviral responses, and inhibiting downstream signal transduction. RNA viruses with small genomes and limited coding space often express multifunctional proteins that modulate several aspects of the normal host response to infection. One such virus, rotavirus, is an important pediatric pathogen that causes severe gastroenteritis, leading to ∼450,000 deaths globally each year. In this review, we discuss the nature of the innate antiviral responses triggered by rotavirus infection and the viral mechanisms for inhibiting these responses.

## Introduction

Viruses interact with the host at all stages of replication—cell entry, viral transcription, translation, genome synthesis and packaging, and cell exit. These interactions are not only crucial for producing new virus progeny, but also enable the host to recognize the presence of an infectious agent. As host species have evolved mechanisms to defend against pathogens, viruses have in turn evolved strategies to avoid the host immune response. For instance, viruses may evade detection by sequestering their own RNAs, perturb the expression and activation of transcription factors required to initiate innate responses, or inhibit downstream signal transduction necessary for amplifying innate or adaptive immune responses [1]. In addition, some viruses specifically suppress cellular mRNA translation, thereby down regulating the expression of certain host proteins with antiviral activity, while simultaneously up regulating selective translation of viral mRNAs [2], [3]. As we discuss below, recent studies have provided new insights into the mechanisms used by the host cell to recognize rotavirus infection and by the virus to avoid recognition and antagonize antiviral pathways.

## Rotavirus Biology

Since their discovery in 1973 [4], rotaviruses have been identified as the most common cause of severe, dehydrating diarrhea in children [5]. Worldwide, rotavirus-related diarrhea results in 453,000 deaths annually in children under 5 years of age [6]. Most deaths attributable to rotavirus occur in low-income countries where there is limited access to the two safe and effective licensed rotavirus vaccines, RotaTeq (Merck and Co., PA, USA) and Rotarix (GSK Biologicals, Rixensart, Belgium) [7].

**Figure 1:**
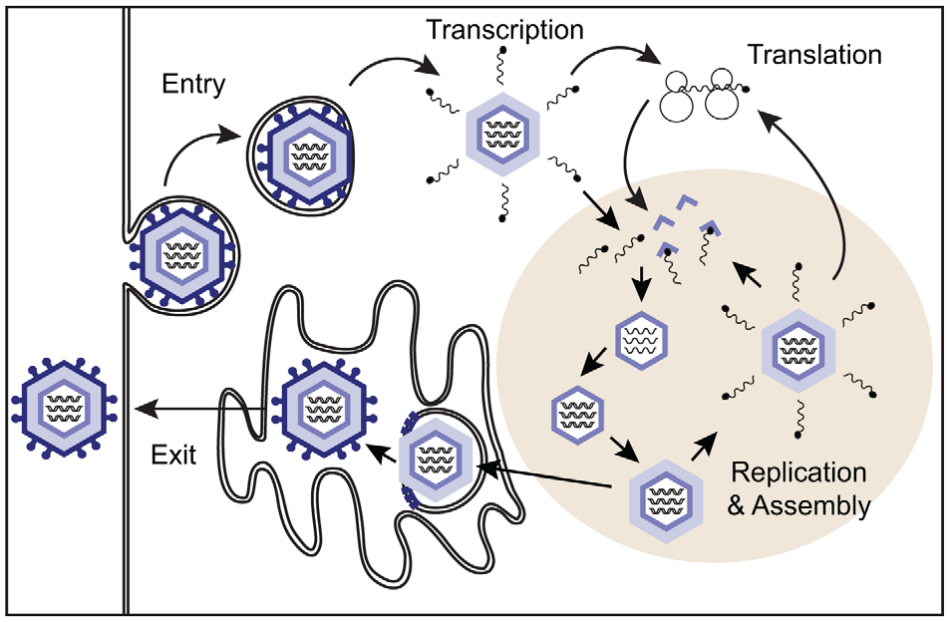
Overview of the rotavirus replication cycle. During entry into the cell, the outermost protein layer of the triple-layered virion is lost. Polymerase complexes in the core of the resultant double-layered particle produce viral mRNAs that are capped but lack poly(A) tails. Viral proteins and RNAs accumulate in protected sites of the cytoplasm called viroplasms where nascent particle assembly takes place. Interaction of newly formed polymerase complexes with the core capsid protein triggers genome replication, which is followed by addition of the intermediate protein layer of the virion. Double-layered particles bud into the endoplasmic reticulum, acquiring their outer capsid. After release through lysis or trafficking, the attachment spike must be cleaved by trypsin-like proteases in the intestinal lumen to activate the virus for subsequent infection.

Rotaviruses, members of the Reoviridae, infect mature enterocytes that cover small intestinal villi and are transmitted by the fecal-oral route [8]. The infectious virion is a non-enveloped, icosahedral particle composed of three concentric protein layers that surround the segmented, double-stranded (ds)RNA genome [9]. The outer capsid of the triple-layered virion is removed during membrane penetration, yielding a double-layered particle that directs viral mRNA transcription via the activity of RNA polymerases contained within the particle's core (Figure 1). Rotavirus mRNAs serve as templates for both protein synthesis and genome replication [10]. Viral mRNAs involved in genome replication accumulate with proteins in electron-dense cytoplasmic inclusions called viroplasms [11]. It is in these inclusions that viral RNAs are packaged and replicated and double-layered particles are assembled [9]. Progeny double-layered particles mature into triple-layered virions by budding into the endoplasmic reticulum. Virions are released by either cell lysis or exocytosis.

## Rotavirus-Triggered Signaling of Pathogen Recognition Machinery

A critical and virtually universal early innate response of the host cell to viral infection is the secretion of cytokines belonging to the interferon (IFN) family, including type I, II, and III IFNs. The secretion of IFN results in the expression of several hundred IFN-stimulated gene (ISG) products with antiviral activities, both within infected cells as well as in bystander populations. The presence of viral RNA in cells infected with RNA viruses is chiefly responsible for triggering the innate immune response, and this response is rapid and inevitable unless the virus has mechanisms for counteracting steps in IFN activation pathways [12].

**Figure 2:**
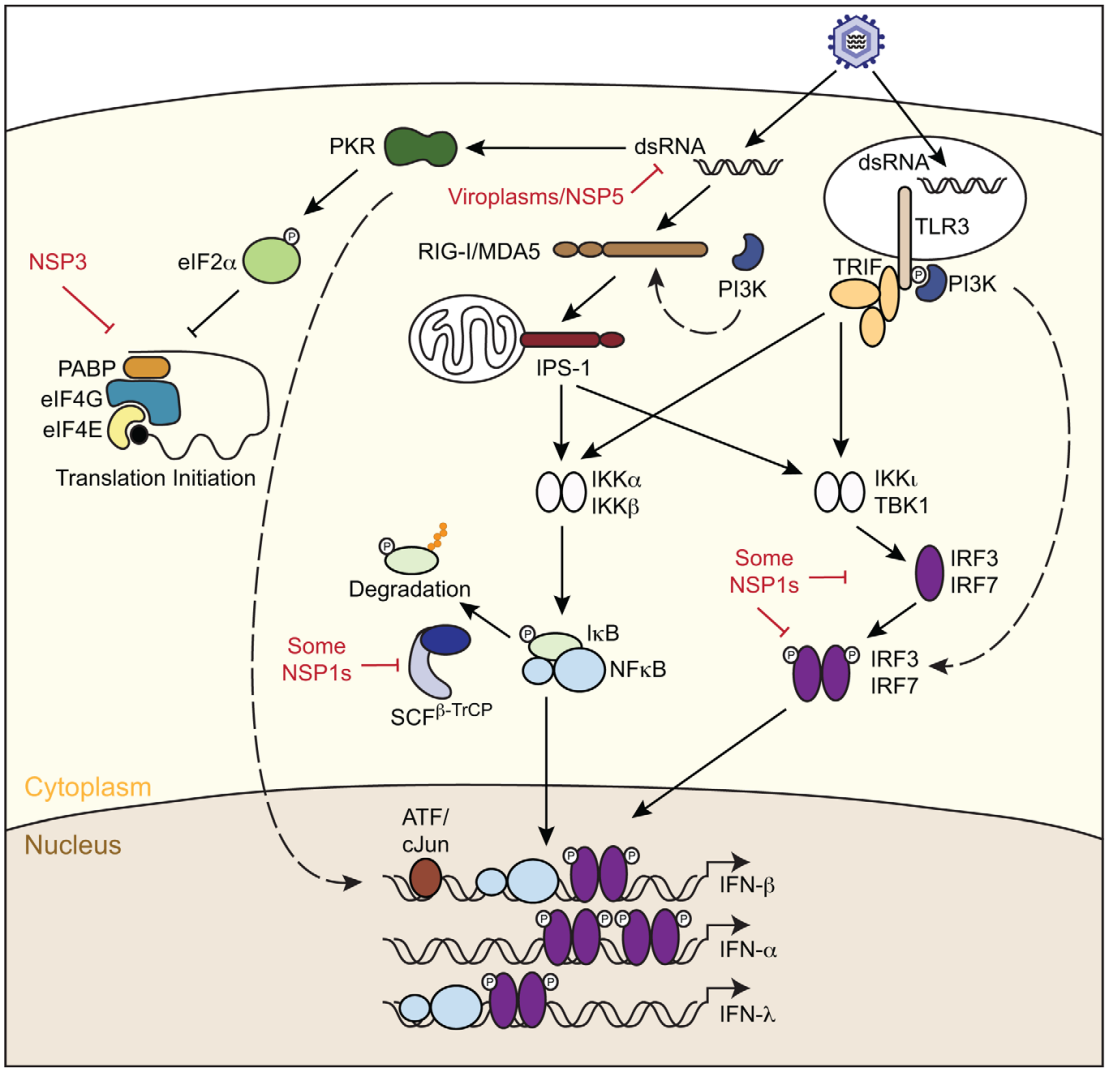
Rotavirus interactions with innate signaling pathways. Viral nucleic acids may be recognized in a host cell by membrane-bound Toll-like receptors (TLR3) or cytoplasmic RIG-I-like receptors (RLRs). When activated by nucleic-acid binding, RLRs recruit and activate the signaling adaptor molecule IPS-1, which recruits a signaling complex that activates latent cytoplasmic transcription factors such as interferon regulatory factor 3 (IRF3) and nuclear factor-κB (NF-κB). TLR3 activation stimulates the recruitment of the adaptor TRIF, which acts as a platform for a variety of signaling molecules that also phosphorylate IRF3 or NF-κB. When signaled, the C-terminus of IRF3 is phosphorylated, causing a conformational change that leads to dimerization and nuclear translocation. NF-κB is held inactive by inhibitor of NF-κB (IκB). Signals generated during viral infection cause phosphorylation of IκB, followed by ubiquitination (orange circles) and proteasomal degradation mediated by the SKP-CUL-F-box-β-TrCP (SCF^β-TrCP^) E3 ubiquitin ligase complex. NF-κB subsequently translocates to the nucleus. IRF3 and NF-κB bind to the IFN-β promoter in a cooperative manner with c-Jun/ATF-2 forming an enhanceosome complex initiating the transcription of IFN-β mRNA. Additional transcription factors, including IRF7, are induced by IFN-β and can also bind to the IFN-β promoter to enhance the transcription of IFN-β and IFN-α genes. PI3K activity may be required for mediating TLR3 and RIG-I signaling by an unknown mechanism (dashed line). PKR responds to dsRNA binding by phosphorylating eIF2α, which ultimately inhibits translation initiation. PKR is also thought to promote the secretion of IFN-β by an unknown mechanism (dashed line). Rotavirus can antagonize innate signaling pathways through several avenues (shown in red), the primary one representing the NSP1-induced degradation of IRF3 and IRF7. Some NSP1 proteins are also known to induce the degradation of β-TrCP. Rotavirus NSP3 can also impede antiviral responses by suppressing the translation of host mRNAs generated from IFN-stimulated genes. By sequestering viral RNAs within viroplasms, the virus can prevent their recognition by PKR, RIG-I, MDA-5, and other sensors that upregulate antiviral responses.

RNA viruses are typically recognized as non-self by the invaded cell through several pattern recognition receptors (PRRs), notably the Toll-like receptors (TLRs), nucleotide-oligomerization domain (NOD)-like receptors (NLRs), and retinoic acid-inducible gene 1 (RIG-I)-like receptors (RLRs) [13]–[15]. These PRRs are differentially expressed as membrane-associated or cytosolic proteins, and recognize pathogen-associated molecular patterns (PAMPs). PAMP–PRR interactions trigger complex signaling cascades that result in the expression of IFN and early antiviral gene products in the infected cell (Figure 2). Secreted IFN binds to IFN receptors to drive autocrine signaling in the infected cell and paracrine signaling in surrounding uninfected cells. Activation of these receptors stimulates the JAK-STAT pathway, which leads to robust ISG expression and establishment of the antiviral state [16].

Rotavirus infection stimulates IFN-β and early antiviral gene expression by a signaling pathway that requires IFN-β promoter stimulator 1 (IPS-1) (also known as MAVS, VISA, or Cardif), an adaptor protein that is recruited to signaling complexes following activation of either of two RLRs: RIG-I or melanoma differentiation-associated protein 5 (MDA-5) [17]–[19] (Figure 2). Interestingly, both RIG-I and MDA-5 are involved in recognizing rotavirus infection and loss of either of these factors substantially decreases the magnitude of IFN-β induction [17], [18]. Inactivated (i.e., non-infectious) rotavirus does not induce early antiviral gene expression in fibroblasts and epithelial cell lines, indicating that a product of viral replication interacts with and activates RLRs in these cell types [18]. Although the exact nature of the rotavirus PAMPs generated during infection of epithelial cells is unknown, current evidence suggests that distinct products of rotavirus replication activate RIG-I and MDA-5 [17], [18], [20]. Identification of the rotavirus PAMPs that are recognized by host RLRs is critical to advancing our understanding of early events in rotavirus–host cell interactions.

Rotavirus recognition by PRRs may be cell type-specific, and other endosomal or surface membrane-associated PRRs such as TLR3, TLR7, and TLR9 have been implicated in stimulating innate responses to rotavirus infection, although more definitive studies on these topics are required [21]–[24]. Unlike the RLRs, the role of specific signaling components downstream of TLRs including the adaptors MyD88 and TRIF (Toll/IL-1 receptor domain-containing adaptor inducing IFN-β) during infection has not been well studied in relevant cell types. TLR7 and/or TLR9 seem to have roles in rotavirus recognition in primary human plasmacytoid dendritic cells (pDCs), a lineage that is only minimally permissive to rotavirus replication [21]. pDCs are a major source of systemic IFN and an important link between innate and adaptive antiviral immune responses [25]. Both replication-competent and inactivated rotaviruses efficiently activate an IFN response in pDCs [21]. The PAMP responsible for this activation appears to be viral genomic dsRNA interacting with TLR7 and/or TLR9 [21]. Although dsRNA is widely recognized as a potent PAMP and purified rotavirus genomic dsRNA induces the expression of IFN-β and other cytokines [26], [27], the rotavirus replication cycle produces protein components that are likely to minimize host cell interaction with the dsRNA genome (e.g., sequestration within the viral particle). Thus, the source and type of RNA recognized by TLRs, as well as whether this interaction involves intra- or extra-cellular TLRs in the infected cell, remain unclear. Limited studies on the role of TLRs during rotavirus infection in the mouse model of infection have noted that MyD88 (mediating TLR7, 8 activation), TRIF (TLR3), or TLR3 are not required for IFN induction in intestinal epithelial cells (IECs) or myeloid DCs (mDCs) following infection with either RRV or murine rotavirus [17], [22]. In contrast, another study reported that age-dependent TLR3 expression is associated with limited rotavirus shedding and type III IFN synthesis, but these effects were specific to adult mice [24]. Further studies are clearly needed to evaluate the role of TLR3 in rotavirus infection, particularly in the primary target, the non-adult host.

Some viruses signal IFN-β production through a protein kinase R (PKR)-dependent pathway, in particular those viruses that activate MDA-5 [28], [29]. During rotavirus infection, PKR does not appear to be involved in the regulation of early antiviral gene induction [18]. However, cells lacking PKR have a profound defect in IFN-β secretion following rotavirus infection despite the accumulation of IFN-β transcripts. Thus, PKR appears to act at a step between IFN-β transcription and secretion. Whether rotaviruses have evolved a specific strategy to undermine this PKR function remains to be explored.

## Rotavirus Regulation of Innate Signal Transduction Pathways

Rotavirus replication is restricted by pretreatment of permissive cells with IFN [30] Likewise, treatment of newborn calves and piglets with IFN prior to rotavirus infection suppresses virus replication and reduces disease severity [31], [32]. Detection of rotavirus infection by host PRRs results in an elevation of type I and II IFNs in children and animals [33]–[35]. Thus, the virus is capable of triggering IFN production and is sensitive to the antiviral effects of ISG expression. Vanden Broecke et al. [36] and Schwers et al. [32] noted that infection of newborn calves with lose doses of rotavirus caused severe but transient diarrhea coinciding with a delay in the production of IFN, whereas animals infected with high doses of virus produced IFN early on and were free of severe diarrhea [36]. These studies suggest that while rotaviruses are susceptible to IFN, they also have mechanisms to avoid or suppress the effects of IFN, at least during the early stages of disease.

In the suckling mouse model of infection, homologous rotavirus strains (i.e., murine) replicate efficiently and cause disease, whereas heterologous strains (i.e., simian, bovine, or porcine) replicate poorly [37]. Replication of some heterologous strains is also restricted in adult wildtype (wt) mice and those lacking either type I or type II IFN receptors [38]. Analysis of reassortant rotaviruses indicates that the viral nonstructural protein NSP1 and virion cell-attachment protein VP4 are the primary determinants of replication restriction in wt mice and mouse cells [37], [39]. In the absence of type I and II IFN receptors or the transcription factor STAT1, suckling mice are deficient in IFN signaling and become much more susceptible to heterologous, but not homologous, rotavirus replication and spread, raising the possibility that the host IFN response, and the capacity of the virus to overcome it, may play a role in limiting intestinal replication and extraintestinal spread, especially of heterologous strains [40]. While both type I and II IFNs are critical for this restriction, suggesting a synergistic role, an unequivocal role for type III IFN in substantially restricting murine rotavirus replication in suckling mice is still lacking. A recent study concluded that mice lacking type III IFN (IFN-λ) receptors are more susceptible to homologous murine rotavirus infection compared to either wt or IFN-αβ-R^−/−^ mice, indicating that type III, rather than type I IFN, is crucial for restraining homologous rotavirus replication in the intestine [41]. Although an important observation, this study used mice lacking only the type I IFN receptor (i.e., IFN-αβ-R^−/−^), rather than IFN-αβγ-R^−/−^ mice, and directly quantified rotavirus shedding in different mouse strains using adult, rather than suckling, mice [41]. Although the authors also quantified murine rotavirus shedding in suckling mice, this experiment was performed 3 days after exogenous administration of purified type III or type I IFN by subcutaneous injection, making it difficult to interpret whether the restriction of replication observed was directly due to effects of type I and III IFN on IECs. Demonstration of an IFN-αβγ-independent, type III IFN-mediated role in restricting homologous rotavirus replication in suckling mice by quantitative measurement of virus replication is thus still lacking. Previously it has been reported that homologous rotavirus replication in suckling mice is only modestly enhanced by the absence of STAT1 (∼10×) [42]. In contrast, heterologous RRV replicates to levels ∼1,000× greater in STAT1-deficient suckling mice compared to their wt counterparts [40]. In our unpublished studies in suckling mice, homologous rotavirus replicates to similar levels in the absence of either types I and II IFN (IFN-αβγ-R^−/−^) or the combined absence of types I, II, and III IFNs (STAT1^−/−^), this level of replication being ∼25-fold higher than wt mice at 16 hpi (AS and HBG, unpublished data). In comparison, the heterologous RRV strain replicates significantly more in both IFN-αβγ-R^−/−^ (∼250× over wt) and STAT1^−/−^ (∼5,000× over wt) hosts. Thus in suckling mice, type III IFNs may play a relatively modest role in restricting the early replication of homologous rotavirus compared to a more potent effect on heterologous strains. Finally, murine rotaviruses have in fact achieved highly efficient replication and pathogenicity in their (wt, non-adult) natural hosts in the presence of an intact innate immune system, and remarkably, only a single infectious dose is needed to initiate infection and disease [37]. As a result, any IFN-mediated restriction of homologous rotavirus is most likely to be modest and incremental, and more substantial effects are likely to be operant on heterologous virus replication, which is highly restricted in the mouse model. Therefore, based on the accumulated evidence, although both IFN-α/β and IFN-λ are likely to play important roles in response to rotavirus infection, their relative contributions may depend on the nature of the rotavirus strain, site of replication, synergistic effects of IFN-γ, early versus sustained replication, and importantly, host age. Elucidating a precise role for IFN in regulating rotavirus replication in vivo is not easily accomplished because of the challenges in measuring and comparing the effects of IFN deficiency on the replication capacity of homologous murine rotaviruses and heterologous non-murine rotaviruses in the gut and systemically in suckling and adult mice.

## Rotavirus IFN-Antagonist NSP1

Following PRR activation in a viral-infected cell, signal transduction can be expected to activate the transcription factors IFN regulatory factor 3 (IRF3) and nuclear factor (NF)-κB, thereby promoting optimal IFN-β expression. However, infection of permissive cell lines with wt rotavirus strains generally does not trigger high levels of IFN-β transcription or secretion, suggesting that these viruses encode proteins that antagonize the IFN-β expression pathway [18], [39], [43], [44]. Indeed, the key protein that inhibits IFN-β expression in rotavirus-infected cells is NSP1, a viral nonstructural protein that has affinity for IRF3 (Figure 2). The interaction of NSP1 with IRF3 stimulates degradation of the transcription factor via a proteasomal-dependent process [39], [43]–[47]. In contrast to infection with wt rotaviruses, infection with mutant rotaviruses encoding C-terminally truncated NSP1 proteins fails to induce IRF3 degradation. As a result, high levels of IFN-β expression occur in these cells [43], [44], [48]. The suppression of IFN-β expression is not mediated solely by the effect that NSP1 has on IRF3, as the protein can also induce the degradation of other members of the IRF family, including IRF5 and IRF7. This capacity of NSP1 to target multiple members of the IRF family reveals that NSP1 is a broad-spectrum antagonist of type I IFN expression in infected cells [48]. Notably, NSP1 proteins of human rotaviruses rely predominantly on the degradation of IRF5 and IRF7 to undermine IFN signaling, while NSP1 proteins of animal rotaviruses tend to target IRF3, IRF5, and IRF7 [48].

There are a few examples where rotavirus strains have been found to express NSP1 proteins that target proteins for degradation, other than those belonging to the IRF family. As an example, the OSU porcine strain of rotavirus inhibits IFN-β expression by inducing the degradation of β-TrCP, an essential protein in the NF-κB activation pathway, rather than IRF proteins [49] (Figure 2). Interestingly, among other rotavirus strains that can induce β-TrCP degradation, some also exhibit IRF degradation activity. Thus, the IRF and β-TrCP targeting activities of NSP1 seem to be functionally distinct, with both capable of suppressing IFN expression [43]. The collective evidence indicates that NSP1 is a multifunctional IFN antagonist, but one whose activities can be quite diverse on IRF and β-TrCP targets depending on the source of the NSP1 (vis-à-vis virus strain) and the cell type used in assays.

NSP1-mediated degradation of IRF proteins and β-TrCP is prevented by the presence of proteasome inhibitors [43], [44], [4]–[49]. Additionally, sequence analysis indicates that a highly conserved RING domain is located near the N-terminus of the NSP1 protein [50]. In combination, these characteristics suggest that NSP1 may function as an E3 ubiquitin ligase, triggering the ubiquitination of IRF proteins and β-TrCP and, as a result, their proteasomal degradation [51]. Definitive experiments necessary to validate this hypothesis include identifying the putative E2 ubiquitin conjugating enzyme that directly interacts with NSP1 and is the source of ubiquitin moities. Intriguingly, NSP1 itself is subject to proteasomal degradation in cells infected by some rotavirus strains and not others [43], [47], [51]. However, the sensitivity of NSP1 to degradation does not always correlate with the protein's ability to degrade IRF3, leaving the biological relevance of the autodegradation-phenotype uncertain.

In addition to its ability to induce the degradation IRF proteins and β-TrCP, NSP1 has other functions that may be connected to antagonizing IFN expression. For instance, near the N-terminus of NSP1 is an RNA-binding domain that allows the protein to specifically recognize the 5′-end of rotavirus mRNAs. This activity might allow NSP1 to mask 5′ features of viral mRNAs that are detected by PRRs [52], [53]. A recent study has indicated that NSP1, including forms of the protein lacking the C-terminal IRF binding domain, can bind to RIG-I and down regulate its PRR activity [54]. Whether this involves the direct interaction of NSP1 and RIG-I, or an interaction that is bridged by RNA or other protein(s), has not been resolved. Nonetheless, the interaction of NSP1 with RIG-I provides an alternative mechanism by which the virus can antagonize IFN induction, one that does not rely on degradation of IRF proteins or β-TrCP [54].

NSP1 had also been found to interact with the P85 subunit of phosphatidylinositol-3 kinase (PI3K), thus activating the antiapoptotic P13K/Akt pathway that favors virus replication [55]–[57]. Although P13K activation is known to have a role in stimulating IRF3 phosphorylation and IFN production in other systems, the effect was not observed in rotavirus-infected cells, perhaps because other NSP1 activities (e.g., IRF degradation) counter the effect. Clearly, further study is required to understand the implications of the interactions between NSP1, RIG-I, and PI3K. Also worth noting is a study showing that rotavirus suppresses IFN signaling by inhibiting STAT1 nuclear accumulation. Although the viral mediator of this effect has not been established, it would seem likely to be NSP1 [58].

## Rotavirus Regulation of Host Translation

It is typical for RNA viruses to encode proteins, like rotavirus NSP1, with activities that are specifically involved in antagonizing one or more critical steps in the IFN signaling pathway [1], [12]. In addition, RNA viruses are noted for their ability to induce changes to the host translational machinery such that the production of viral proteins is favored and the production of cellular proteins is inhibited [59]. This characteristic allows viruses, including rotavirus, a second, more broadly based mechanism of overcoming the antiviral effects of the IFN signaling pathway by impeding the expression of the many host ISG products that could interfere with productive virus replication.

Efficient translation of most cellular mRNAs requires the participation of both the 5′ cap and the 3′ poly(A) tail and the formation of a cap-initiation complex. By relying on atypical mRNAs and modifying the need for the prototypic cap-initiation complex, viruses can bypass cellular translation mechanisms in order to promote their own protein production in the face of host translational shut-down [2], [3]. Ribosome recruitment to cellular mRNAs is mediated by eukaryotic initiation factor (eIF) 4F, a large protein complex that has affinity for the 5′-cap [60]. The eIF4F-mRNA complex recruits eIF4G, a central adapter protein that binds a number of translation factors and regulators, including poly(A)-binding protein (PABP). The interaction of eIF4G with PABP is essential for the initiation of cellular mRNA translation, and by directing mRNA circularization, is believed to promote ribosome recycling in polysomes [60]. While both cellular and rotavirus transcripts contain similar 5′ cap structures, rotavirus transcripts contain a highly conserved 3′ consensus sequence instead of a poly(A) tail [61]. Rotavirus inhibits cellular mRNA translation through the activities of its nonstructural protein NSP3, which binds to eIF4G and disrupts its interaction with PABP [62] (Figure 2). NSP3 also stimulates PABP accumulation in the nuclei of infected cells, suggesting that NSP3 might interfere with shuttling of nascent cellular mRNAs from the nucleus to the cytoplasm [63]–[65]. NSP3 specifically recognizes and binds to the 3′ consensus sequence of rotavirus transcripts [66], [67]. By binding both eIF4G and the 3′ consensus sequence, NSP3 may promote the circularization of rotavirus mRNAs while sabotaging host translation [62], [68]. Consistent with this model, purified NSP3 alone does not have an effect on in vitro poly(A) mRNA translation. Instead, the presence of viral mRNA is required for inhibition of poly(A) mRNA translation, suggesting the affinity of NSP3 for the translational machinery is changed by its binding to viral mRNA [69]. However, the model for the role of NSP3 in promoting viral translation and inhibiting host translation may be incomplete, given recent results showing that siRNA-mediated knockdown of NSP3 expression in infected cells neither prevents viral protein expression nor reduces virus yields [70]. Similarly, a rotavirus mutant that encodes a defective NSP3 has been found to express wt levels of viral protein in infected cells and to grow to high titers [63].

The phosphorylation status of eIF2α also plays a role in the shutoff of cellular protein synthesis during rotavirus infection [65]. Cells respond to stress and changing growth conditions through eIF2α phosphorylation, which mediates binding of the initiating Met-tRNA to the ribosome. Rotavirus infection induces eIF2α phosphorylation in a PKR-dependent manner, resulting in inhibition of cellular, but not viral, translation [65], [71]. Although siRNA-mediated knockdown of PKR in rotavirus-infected cells prevents eIF2α phosphorylation, viral translation and replication are unaffected [65], [71]. It has been suggested that eIF2α phosphorylation is mediated by the interaction of rotavirus dsRNA with PKR [71]. If so, this raises the interesting question as to the source of the dsRNA, since current models of rotavirus replication do not propose that naked viral dsRNA is produced within the infected cell. Nonetheless, studies carried out with anti-dsRNA antibody suggest that infected cells may contain some amount of either dsRNA or highly structured single-stranded RNA [71], thus providing a possible source of RNA that could be recognized by PKR and trigger eIF2α phosphorylation or that could be recognized by host PRRs and activate IFN production. An important unresolved question is whether the naked RNA represents an intermediate molecule in the viral replication cycle or perhaps an accumulating dead-end by-product.

## Rotavirus Sequestration of Replication Machinery

The replication process of RNA viruses results in the production of numerous copies of viral RNAs and replication intermediates (RIs) that have the potential to trigger activation of IFN signaling pathways. Viruses can counter such activation by sequestering the RNAs through interactions with viral RNA-binding proteins, packaging the RNAs within progeny capsids, or limiting the accumulation of the RNAs to specialized cytoplasmic inclusion bodies. In the case of rotaviruses, packaging and replication of the viral dsRNA genome take place in cytoplasmic inclusion bodies (viroplasms) that are rich in viral RNA and protein [11]. Studies with siRNAs indicate that viroplasms are “safehouses” in which viral RNAs are protected against RNAi degradation pathways [72]. The RNA-sequestration phenotype may be mediated by the abundance of RNA-binding proteins within the viroplasm, the most notable being the nonstructural RNA-binding protein NSP2 [73] and the core capsid protein VP2 [74]. Other viral RNA-binding proteins in the viroplasm may contribute to RNA sequestration, although they have weaker affinity for RNA (NSP5) [75] or exist in low levels (RNA polymerase VP1, RNA-capping enzyme VP3) [76], [77]. The presence of RNA-binding proteins probably interferes with recognition of the viral RNAs in viroplasms by RIG-I and MDA5, suppressing IFN induction. Existing data suggest that as rotavirus dsRNAs are synthesized, they are concurrently packaged into pre-virion cores, thus avoiding the production of naked dsRNA [78], [79]. This packaging-linked replication mechanism can interfere with dsRNA recognition by PRRs or PKR that would up regulate IFN expression. Together, these characteristics suggest that sequestration of viral RNAs in the viroplasm and during genome replication are important factors in delaying the establishment of an antiviral state in rotavirus-infected cells.

## Conclusions and Perspectives

Rotavirus infection generates 11 viral dsRNAs and 11 mRNAs, creating an intracellular environment rich in potential PAMPs recognizable by RIG-I, MDA5, TLRs, and PKR [80], [81]. Yet, rotavirus-infected cells are generally characterized by low levels of IFN expression. The suppression of the IFN response can be connected to number of viral mechanisms, including sequestration of viral RNA in viroplasms and dsRNA in virus particles, interaction of NSP1 with RIG-I, NSP1-induced degradation of IRF proteins and/or β-TrCP, and prevention of STAT1 activation. IFN suppression and establishment of the antiviral state is likely also mediated by the virus's capacity to down regulate host translation via its NSP3 protein.

The only rotavirus protein with a clearly established specific role in down regulating IFN expression is NSP1. A chief mechanism of NSP1 action seems to be to induce the degradation of multiple members of the family of IRF proteins, including those involved in IFN-α and -β expression. The characteristics of NSP1, particularly the presence of an N-terminal RING domain, suggest that the protein functions as an E3 ubiquitin ligase. Identifying the E2 ubiquitin conjugating enzyme that works in concert with NSP1 may help to explain why some NSP1 proteins fail to induce degradation of targets in different cell types. Perhaps, some cell types lack the appropriate E2 enzyme to support NSP1 function. The target of NSP1-mediated ubiquitination on IRF targets has not been resolved, but presumably is one that is shared by at least some members of the IRF family (IRF3, IRF5, IRF7). The fact that NSP1 cannot induce degradation of all members of the IRF family (e.g., IRF1) emphasizes that the activity of NSP1 is specific and targets only a subset of IRF proteins.

Several rotavirus proteins, including the enterotoxin NSP4, host translational inhibitor NSP3, and RNA capping enzyme VP3, have been implicated in influencing growth, spread, and virulence of viruses in different animal species [82]–[84]. NSP1 also plays a critical role in replication, virulence, and extraintestinal spread [37], [40]. More studies are needed to directly examine the role of NSP1 and other viral proteins in promoting the ability of homologous, as opposed to heterologous, rotaviruses to replicate efficiently in the host intestine. While studies have identified NSP1 and its role in counteracting IFN-mediated antiviral responses as critical components of extraintestinal biliary tract replication and disease, there is a relative lack of information concerning the role of innate immunity in controlling local versus systemic phases of rotavirus replication in most animal species [40], [85]. In addition, NSP1 or other rotavirus proteins may impact innate immunity-mediated restriction of replication or virulence in ways not yet understood.

Studies with other viruses have shown that addition of a fully methylated cap to viral mRNAs promotes escape from recognition by ISG56 and ISG54 (also known as IFIT1 and IFIT2, respectively) and masks exposed 5′-phosphate moieties that trigger RIG-I [86], [87]. Methylation of the RNA cap by rotavirus VP3 may thus serve to subvert the host innate antiviral response through avoidance of PRR activation and/or ISG-mediated suppression. Conversely, differences in the capping efficiency of VP3 among rotavirus strains may constitute a virulence determinant, and is an unexplored mechanism by which rotavirus may further modify the innate immune response. Interestingly, it has been noted that an IFN-activating rotavirus strain can inhibit the poly(I:C)-directed transcriptional activity of IRF3, despite its failure to degrade IRF3 [47]. Thus, additional means of IFN inhibition by rotavirus may exist. A more comprehensive understanding of the in vivo ISG and IFN responses to homologous and heterologous rotavirus strains in different cell types and organs should lead to identification of additional rotavirus proteins and host factors that are involved in species- and tissue-specific viral replication restriction and pathogenesis.
